# Effect of neurotransmitters and bone marrow cells for neuronal regeneration in iatrogenic spinal cord injury: An experimental study

**DOI:** 10.4103/0019-5413.65142

**Published:** 2010

**Authors:** PS John, CS Paulose, R Sreekanth

**Affiliations:** Department of Orthopedics, Medical College, Kottayam, Kerala, India

**Keywords:** Spinal cord injury, neurotransmitters, bone marrow cells

## Abstract

**Background:**

Spinal cord trauma is a major health problem with associated physical, social, economic and psychological sequelae. Despite many advances in research and treatment modalities, the pathophysiology of spinal cord injury remains unclear, and morbidity and mortality among these patients remain high. This experimental study investigates the regenerative cell proliferation effects of bone marrow supplemented with neurotransmitters combinations in the regeneration of spinal cord injury

**Materials and Methods:**

Ethical Committee Clearance was obtained for animal study. All animal care and procedures were in accordance with the CPCSEA and National Institute of Health guidelines. Thirty Wistar rats with monoplegia following surgical hemitransection of the spinal cord were used for the study. Half of them were randomly selected as the test group and the rest as the control group. Spinal cord injury model of Wistar rats in the test group were treated by infusing a combination of neurotransmitters and bone marrow at the site of injury using a special polythene tube and reservoir for 21 days. In the control group of rats with monoplegia, normal saline was infused at the site of injury for 21 days. The observations are recorded along with results.

**Results:**

The monoplegia in the test group of rats recovered significantly (*P* value < 0.01) with supplementation of the bone marrow cells and neurotransmitters combination. In the control group of rats, there was no recovery. The reward-seeking locomotor test and sensory recovery test confirmed recovery from spinal cord injury in the test group with significance.

**Conclusions:**

The neurotransmitters and bone marrow combination was responsible for functional recovery in the test group of rats with experimental spinal cord injury We believe that the combination of neurotransmitters along with bone marrow may be a scope of future research in patients with spinal cord injury.

## INTRODUCTION

Spinal cord trauma is a major health problem with accompanying physical, social, economic and psychological sequelae. It affects mainly young adults, and the incidence of spinal cord injury is estimated to be between 35 and 80 per million population per year in most nations.^1^–^3^ Despite many advances in research and treatment modalities, the pathophysiology of spinal cord injury remains unclear, and morbidity and mortality among these patients remains high. Restoration of locomotor function after damage to the spinal cord is an enormously challenging problem. Traumatic spinal cord injury has profound implications for the patient and the medical–social support system as well. Following spinal cord injury, significant re-organization of sensorymotor pathways occur caudal to the lesion.^3^ The major difficulty is that the axons of the damaged neurons do not regenerate in the spinal cord under normal conditions, unlike axons in the peripheral nervous system (PNS), which have the capacity to regrow.^1^ After spinal cord injury, several inhibitory extracellular matrix molecules combine with reactive astroglia at the lesion site form a dense scar that acts as a barrier to regenerating axons.^1^ Consequences of spinal cord injury are devastating and any strategy to mitigate neurological loss is attractive.^2^

Scientists have demonstrated nerve cells outside the brain and spinal cord can regenerate.^1^ These experiments encouraged the idea that adult nerve cells in the spinal cord can proliferate and re-establish the connections in an appropriate growth environment.^2^,^3^ Neurotransmitters relay, amplify and modulate signals between the neurons. Serotonin (5HT) and gamma-aminobutyric acid (GABA) acting through specific receptor subtypes 5HT_2_^4^ and GABA_A_, _B_^5^, respectively, control cell proliferation as comitogens. Neurotransmitters’ integration into biodegradable polymers result in a biomaterial that successfully promotes nerve growth, which is necessary for victims of central nervous system (CNS) injury, stroke or certain neurodegenerative diseases to recover sensory, motor, cognitive or autonomic functions. GABA receptors are involved in early events during neuronal development. The presence of GABA receptors in developing oligodendrocytes provides a new mechanism for neuronal–glial interactions during development and offers a novel target for promoting remyelination following white matter injury.^6^ Metabotropic glutamate receptors modulating neuronal development and survival have been identified in oligodendrocyte progenitor cells. Findings in many studies have shown the cell-specific effect of serotonin on regenerating neurons within the adult CNS by increasing the calcium concentration of the cells.^7^

One approach for repairing spinal cord injuries in animals is by transplanting cells or pieces of peripheral nerves that produce substances that create an environment for axons to grow. It is suggested that implanting cells from the PNS into the area of a CNS injury helps neuronal regeneration. Because the environment of the PNS supports axon regeneration, it is believed that recreating this environment in the spinal cord allows CNS axons to regenerate after an injury. Ideally, this environment would also direct the growing nerves to the correct targets.^8^ Research supporting the preclinical studies to assess the safety, feasibility and efficacy of implanting autologous bone marrow stem cells into spinal cord injury was performed in patients.^9^,^10^

Regeneration is also carried out by fetal tissue implantation containing stem cells, progenitor cells and growth factors, which promote axonal growth.^3^ Stem cells can differentiate into various cell types, depending on the signals they receive. Fetal tissue transplantation into spinal cord with the right chemical signals help them to develop into neurons and supporting cells, re-establishing lost circuits.^6^–^8^ Bone marrow cells have been shown to actively remyelinate spinal cord once administered directly or intravenously.^10^ Combination of neurotransmitters as therapeutic agents for cell proliferation and differentiation is important in spinal cord regeneration. Up-regulation of GABA and 5HT receptor subtypes were reported in accelerated fracture healing.^11^ This study investigates the effect of regenerative cell proliferation in regeneration of spinal cord injury, when bone marrow supplemented with combination of neurotransmitter are given.

## MATERIALS AND METHOD

**Figure 1:**
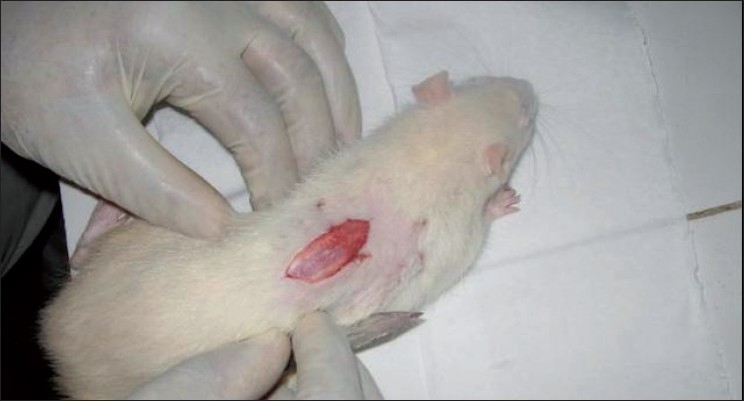
Clinical photograph depicting exposure of the spinal cord at the level of T_12_ vertebra is experimental rat

**Figure 2:**
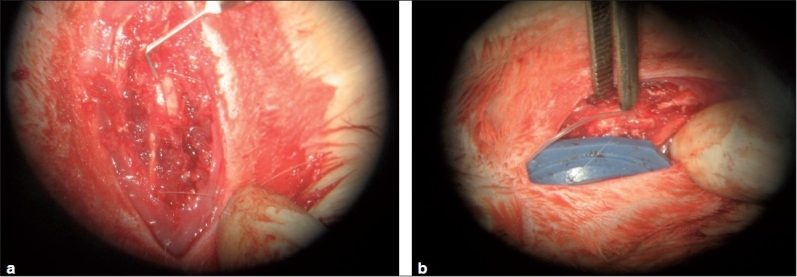
(a) Intraoperative photograph depicting hemisection of the spinal cord (b) *In situ* placement of specially designed ruber chamber (reservoir) with tube

Ethical Committee Clearance was obtained for animal study. We conducted our study in 6-month-old male Wistar rats. Thirty rats, 15 each in the test group and control group were used for the study. All the animals were housed in separate cages under 12-h light and 12-h dark periods and were maintained on standard food pellet and water. All animal care procedures were in accordance with the institutional CPESCA guidelines. Under all aseptic precautions and ether anesthesia, monoplegia was induced by performing a laminectomy and hemitransection of the cord under mangnifying loupe at the level of the 12^th^ thoracic vertebra of the experimental rats [Figures 1 and 2a].

A specially designed rubber chamber with silastic tube^12^ was inserted subcutaneously, with the tip of the silastic tube inserted at the injury site and fixed with sutures. The rubber chamber at the other end of the tube was buried under the skin during the time of wound closure [Figure 2b]. Only local antibiotics for wound care was used. This special silastic tube with a chamber attached on one end was developed in the Department of Polymer Science, Cochin University of Science and Technology, Cochin^12^. Spinal cord injury was confirmed by monoplegia. After 3 weeks, these rats with monoplegia were divided into test and control groups (15 in each group). Then, the control group was given physiological saline solution through the chamber daily for 21 days. The test group was given bone marrow through the chamber on the first day of treatment and a combination of GABA and serotonin (1 *μ*g/kg body weight) through the chamber for 21 days. Bone marrow was aspirated from the supracondylar area of the femur by a special technique. Ten drops of normal saline were injected into the femur at the supracondylar area and the marrow washing was aspirated for injection into the chamber. The neurotransmitter combination was injected into the reservoir from which it drained down through the silastic tube into the site of spinal cord injury.

**Figure 3:**
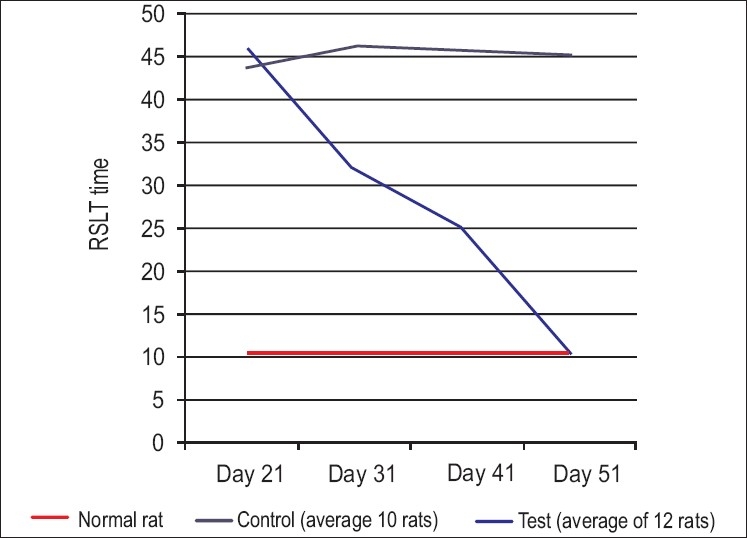
Graph depicting reward seeking locomotor test in the test and control groups and normal rats

Spinal cord regeneration and re-establishment of connections were^13^,^14^ assessed by the motor recovery-reward-seeking locomotor test.^17^,^20^ Rats were deprived of food for 24 h continuously after which a source of food was kept at a distance of 1 m in a special unidirectional pathway. The time taken by the rat to reach the food source from this 1-m distance was taken as an indirect measurement of motor recovery.The reward seeking lokomotor test was done on 21^st^, 31^st^, 41^st^ & 51^st^ days [Figure 3].

## RESULTS

Of the 30 rats studied (15 cases and 15 controls), three among the test group and five among the control group died within first week after surgery. There was no further mortality in the test groups after supplementation of neurotransmitters and bone marrow. Six of the control group rats had died after 2 months and the recovered rats had a fairly normal life time. Only those rats in both groups which survived the whole period of study were included for analysis.

Thus, 12 rats in the test group and 10 rats in the control group were analyzed by the reward–seeking locomotor test on days 21, 31, 41 and 51 for their motor recovery and by withdrawal reflex for sensory recovery. All the rats in the test group showed remarkable (motor and sensory recovery of hind limbs, but the speed and strength was less than normal, as quantified by the reward-seeking locomotor test) improvement by an average time period of 14 days and continued to improve to full recovery by 28 days. Rats in the control group, which were supplemented with normal saline, did not show much improvement [Figure 3].

## DISCUSSION

Traumatic spinal cord injury (SCI) typically results in axonal damage and cell death, leaving individuals with varying degrees of functional impairments. The extent of these impairments is dependent on both the severity of the injury as well as the level at which the injury occurred. Damage arising from acute SCI is generally described as two distinct pathophysiological events. The damage incurred at the time of injury is termed primary injury and typically results from direct mechanical disruption of cord integrity. In contrast, secondary injury occurs in a delayed, yet progressive, fashion and involves cellular and biochemical events that initiate cascades culminating in tissue damage and cell death.^25^,^15^ It is well recognized that the evolution of secondary tissue damage spreads away from the injury epicenter, incorporating tissues both rostral and caudal to the primary lesion with increasing functional deficits. All patients who had spinal cord damage would eventually become patients with varying degrees of persisting neurological deficit, unless some recovery occurs, which, to our knowledge, is unpredictable.^3^,^16^ Neuroprotective strategies aimed at preventing damage arising from secondary injury processes provide some hope for tissue sparing and improved functional outcome.^3^ This, in conjunction with the fact that current treatment options are limited, hastens the need to find novel therapeutic agents.

The present study has tested whether bone marrow and neurotransmitter modulation of the local proteoglycan-rich milieu at the injury site would create an environment more conducive to the regeneration of axons after experimental hemitransection of the spinal cord. The reward-seeking locomotor test revealed significant improvement in hind limb function in the bone marrow and neurotransmitter-treated group compared with the control animals. Pittenger *et al*.^17^ reported that mesenchymal stem cell derived from the bone marrow differentiated into osteocytes, chondrocytes and adipocytes. The multipotent adult progenitor cells derived from the bone marrow,^18^ which comprise approximately 0.125% of the total marrow cells,^19^ are multipotent stem cells with the capacity to differentiate, under specific experimental conditions,^13^ into several different types of cells, including osteoblasts, adipocytes, chondrocytes, skeletal muscle fibers, cardiomyocytes, hepatocytes, neural cells and epithelial cells of the lung and intestinal tract. It has recently been reported that the bone marrow-derived cells also have the potential to develop into neural lineages, such as neurons and astrocytes, both *in vivo*^20^ and *in vitro*.^21^ Bone marrow cells that are adherent in the culture of bone marrow aspirates have already been used for the treatment of the injured spinal cord^14^ and brain. The transplantation of bone marrow cells by direct injection into the lesion might promote tissue repair in the injured spinal cord by reducing the size of the cavity at the lesion. The effects of transplanted bone marrow cells on tissue repair, as described above, suggest that some trophic factors might be released from bone marrow cells to promote the tissue repair.^22^

Neurotransmitters relay, amplify and modulate signals between neurons. Serotonin (5HT) and GABA, acting through specific receptor subtypes 5HT_2_ and GABA_A_, _B_, control cell proliferation as comitogens.^4^,^5^ Integration of neurotransmitters into biodegradable polymers results in a biomaterial that successfully promotes nerve growth, which is necessary for victims of CNS injury, stroke or certain neurodegenerative diseases to recover sensory, motor, cognitive or autonomic functions. GABA receptors are involved in early events during neuronal development. The presence of GABA receptors in developing oligodendrocytes provides a new mechanism for neuronal–glial interactions during development and offers a novel target for promoting remyelination following white matter injury^6^. Metabotropic glutamate receptors modulate neuronal development and survival and have been identified in oligodendrocyte progenitor cells. Findings showed the cell-specific effect of serotonin on regenerating neurons within the adult CNS of the pond snail by increasing the calcium concentration of the cells.^7^ The combination of neurotransmitters as therapeutic agents for cell proliferation and differentiation has importance in spinal cord regeneration. Up-regulation of GABA and 5HT receptor subtypes were reported in accelerated fracture healing.^23^–^25^

In these experiments, we found that neurotransmitters and bone marrow cells facilitated significant recovery from SCI in rats. This was true for both hind limb function and sensory recovery. Immediately following SCI, both groups exhibited severe deficits in hind limb function and locomoted using only the forelimbs. There was no significant recovery in the saline group. In contrast, neurotransmitter and bone marrow-treated rats showed continued and significant improvements over the recovery period. Promising results in our study show that treatment with neurotransmitters and bone marrow can facilitate functional recovery and axonal regeneration in spinal cord injury. The exact mechanism by which neurotransmitters and bone marrow facilitate spinal cord regeneration needs further research. It is proposed that the particular combination of neurotransmitters used in this study modulates the bone marrow cells into neurons for regenerating the surgically transectioned spinal cord of the rat model. Similar strategy for regeneration of spinal cord injury has never been mentioned in the literature so far. If we can effectively utilize the same treatment strategy in clinical trials, It will be a great breakthrough in the management of patients with chronic spinal cord injury.
